# Comparing utility functions between risky and riskless choice in rhesus monkeys

**DOI:** 10.1007/s10071-021-01560-x

**Published:** 2021-09-27

**Authors:** Philipe M. Bujold, Leo Chi U. Seak, Wolfram Schultz, Simone Ferrari-Toniolo

**Affiliations:** Department of Physiology, Development and Neuroscience, University of Cambridge, Cambridge, CB2 3DY UK

**Keywords:** Gamble, Preference, Prospect theory, Economic choice, Decision-making

## Abstract

Decisions can be risky or riskless, depending on the outcomes of the choice. Expected utility theory describes risky choices as a utility maximization process: we choose the option with the highest subjective value (utility), which we compute considering both the option’s value and its associated risk. According to the random utility maximization framework, riskless choices could also be based on a utility measure. Neuronal mechanisms of utility-based choice may thus be common to both risky and riskless choices. This assumption would require the existence of a utility function that accounts for both risky and riskless decisions. Here, we investigated whether the choice behavior of two macaque monkeys in risky and riskless decisions could be described by a common underlying utility function. We found that the utility functions elicited in the two choice scenarios were different from each other, even after taking into account the contribution of subjective probability weighting. Our results suggest that distinct utility representations exist for risky and riskless choices, which could reflect distinct neuronal representations of the utility quantities, or distinct brain mechanisms for risky and riskless choices. The different utility functions should be taken into account in neuronal investigations of utility-based choice.

**Supplementary Information:**

The online version contains supplementary material available at 10.1007/s10071-021-01560-x.

## Introduction

Whether we are choosing between fruits or vegetables at the supermarket, deciding to jaywalk in the face of incoming traffic, or picking the ideal friends to go traveling with, most of our decisions fall under two categories: some have certain outcomes, some do not. Economists call these decisions risky and riskless, respectively. Different from everyday definitions that often view risk as probability of loss or damage, ‘risk’ in economics refers to higher statistical moments of probability distributions of choice outcomes, in particular variance, skewness and kurtosis (Rothschild and Stiglitz 1970; d’Acremont and Bossaerts 2016; Genest et al. 2016).

In economics, Expected Utility Theory (EUT) (von Neumann and Morgenstern 1944) served as the dominant model of risky decision-making until the inception of behavioral economics in the 1970s. Under EUT, a decision-maker’s attitude towards risk was fully captured by the curvature of their utility function: a mapping of reward quantities onto an internal, subjective metric. A concave utility function predicted an aversion to risk, while a convex one predicted risk-seeking behavior. Being a mathematical representation of economic preferences (Kagel et al. 1995), utility is a fundamental decision variable, and it is therefore reasonable to assume that a single function might be common to all forms of economic choice. Thus, a risky utility function estimated according to EUT should also be valid for riskless choice. However, experimental evidence indicates discrepancies between risky and riskless utility functions (Barron et al. 1984; Stalmeier and Bezembinder 1999).

Contrasting with EUT, Prospect Theory (PT) highlights an important difference between risky and riskless choice through the introduction of subjective probability weighting. As probability weights the utility of each outcome for computing Expected Utility, empirical tests have shown that this weight may not derive from the true, 'physical' probability but is distorted at specific probabilities in humans (Kahneman and Tversky 1979; Tversky and Kahneman 1992; Gonzalez and Wu 1999) and monkeys (Stauffer et al. 2015; Ferrari-Toniolo et al. 2019). Thus, rather than being solely predicted by an individual’s utility curvature, one’s risk attitude would also vary with their subjectively weighted outcome probabilities. In other words, while EUT assumed that risk attitudes derived exclusively from the way in which people value rewards as reflected in the curvature of the utility function, PT made the case for an additional subjective weighting of probability.

Subjective probability weighting has been widely incorporated into studies of risky and riskless decision-making (Kahneman et al. 1990; Lattimore et al. 1992; Camerer et al. 2002; Hertwig and Erev 2009). With all the studies on behavior that use subjective probability weighting, there is a remarkable lack of research validating its predictions in both risky and riskless choices; the limitation being that risky utilities (or PT values) are usually measured from choices between risky options in humans (Stott 2006; Tversky and Kahneman 1992) and monkeys (Stauffer et al. 2014; Genest et al. 2016), while this clearly cannot be done in a riskless context. One interesting avenue has been to compare risky and riskless preferences via introspective metrics. In a study by Stalmeier and Bezembinder (1999), medical patients were asked questions that involved risky outcomes:”would you rather: live 20 years with a migraine on x days per week (followed by death), or live 20 years with a *p*% chance of getting migraines y times a week, *z* times a week otherwise”; and questions where all options were riskless:” which difference is larger: the difference between 0 days of migraine and x days of migraine, or the difference between x days of migraine and 3 days of migraine”. Modelling with inclusion of subjective probability weighting resulted in identical risky and riskless utilities, and that probability weighting accounted for most of the discrepancy between the risk attitudes measured from risky choices and the risk attitudes predicted by riskless utilities. A similar approach using money outcomes (gains) rather than medical outcomes (losses) led to a similar conclusion: PT successfully reconciled risky and riskless utilities (Abdellaoui et al. 2007).

As the human participants in these studies were generally risk-averse (for gains), inclusion of subjective probability weighting might also reconcile risky and riskless utilities for risk-seeking decision-makers. Additionally, the results of these introspective studies have recently been challenged by a set of studies using a more modern, incentive-compatible approach: the use of time trade-offs as means to study riskless decisions (Cheung 2016). In these studies, humans made choices between larger rewards delivered in the future (with certainty) and smaller rewards delivered now; utilities from intertemporal choices were then compared to those estimated from risky choices. Unlike introspective experiments, however, most research on time trade-offs reports discrepancies between riskless, time-discounted utility functions and risky ones (Andreoni and Sprenger 2012; Abdellaoui et al. 2013; Lopez-Guzman et al. 2018, but see Andersen et al. 2008). These discrepancies cannot even be resolved by probability weighting, which is not entirely surprising given that temporal aspects add an additional factor to utility estimation.

The lack of clear insight into the inclusion of subjective probability weighting to reconcile risky and riskless choices represents a crucial limitation to the interpretation of PT, particularly as it rapidly became the de facto model of choice to study behavior and neuro-economics in humans, monkeys and rats (De Martino et al. 2006; Lakshminarayanan et al. 2011; Marshall and Kirkpatrick 2013; Stauffer et al. 2015; Chen and Stuphorn 2018; Farashahi et al. 2018; Ferrari-Toniolo et al. 2019). Simultaneously, since there have been no attempts at reconciling risky and riskless utilities in non-human decision-makers, there is no evidence to suggest that either human interpretations can be used to explain animals’ choice behavior.

The aim of the present study was to compare risky and riskless utilities in rhesus macaques. Studies on monkeys are highly relevant to humans, as they belong to an evolutionary close species, have distinguished behavioral capacities, possess sophisticated brain functions, and allow fine-grained neuronal analysis of behavioral relationships that are difficult to obtain in humans. Specifically, monkeys show similar economic choice behavior as humans, such as risk preferences (McCoy and Platt 2005; Strait and Hayden 2013; Stauffer et al. 2014), compliance with first-, second- and third-order stochastically dominating gambles (Genest et al. 2016) and well-ordered preferences for multi-component options (Pastor-Bernier et al. 2017). Given the suitability of monkeys for neurophysiological studies, we respected the required control of sensory and motor variables and tested the animals in a well-defined primate laboratory environment rather than in holding cages or the wild. Rather than testing the full spectrum of risk choices incurred in everyday life, we selected specific risk tests as behavioral tools for eliciting risky and riskless utilities. We therefore tested monkeys in two categorical types of binary choice: risky choice between one certain (riskless) and one uncertain (risky) juice reward option, and riskless choice between two certain (riskless) juice magnitudes. We estimated utility functions statistically from empirically assessed choices using the PT-based discrete choice model (Tversky and Kahneman 1992) in combination with the random utility maximization (RUM) model (McFadden 1974, 2001). Importantly, this risky/riskless design addressed two of the most important caveats in human studies: (i) both risky and riskless choices were incentive-compatible (relying on revealed preferences rather than introspection), and (ii) choice options were presented in the exact same way for both risky and riskless decisions.

This study builds on previous human studies that had shown closer similarities between risky and riskless utilities once probability weighting had been accounted for. Therefore, we parametrically separated the contributions of utility and probability weighting on the monkeys’ risky choices. We did not expect identical utilities and even hypothesized that two different utility quantities or mechanisms could drive behavior in risky and riskless choices.

## Animals, materials and methods

### Ethical note

This research has been ethically reviewed, approved, regulated, and supervised by the following institutions, committees and individuals in the UK and at the University of Cambridge (UCam): the Minister of State at the UK Home Office, the Animals in Science Regulation Unit (ASRU) of the UK Home Office implementing the Animals (Scientific Procedures) Act 1986 with Amendment Regulations 2012, the UK Animals in Science Committee (ASC), the local UK Home Office Inspector, the UK National Centre for Replacement, Refinement and Reduction of Animal Experiments (NC3Rs), the UCam Animal Welfare and Ethical Review Body (AWERB), the UCam Governance and Strategy Committee, the Home Office Establishment License Holder of the UCam Biomedical Service (UBS), the UBS Director for Governance and Welfare, the UBS Named Information and Compliance Support Officer, the UBS Named Veterinary Surgeon (NVS), and the UBS Named Animal Care and Welfare Officer (NACWO).

### Animals

**Fig. 1:**
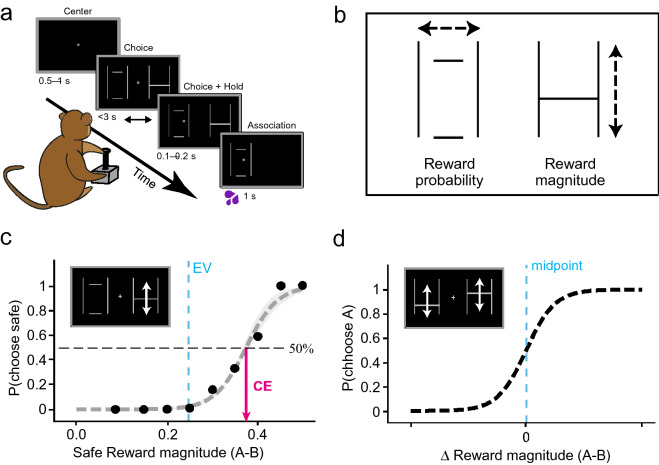
Experimental design and measures of risky and riskless choices. **a** Binary choice task. The monkeys chose one of two gambles with a left–right motion joystick. They received the blackcurrant juice reward associated with the chosen stimuli after each trial. Time, in seconds, indicate the duration of each of the task’s main events. **b** Schema of visual stimuli. Rewards were visually represented by horizontal lines (one or two) set between two vertical ones. The vertical position of these lines signalled the magnitude of said rewards. The width of these lines, the probability that these rewards would be realized). **c** Estimating certainty equivalents from risky choices. Monkeys chose between a safe reward and a risky gamble on each trial. The safe rewards alternated pseudo-randomly on every trial—they could be of any magnitude between 0 and 0.5 ml in 0.05 ml increments. Each point is a measure of choice ratio: the probability of choosing the gamble option over various safe rewards. Psychometric softmax functions (Eq. 1) were fitted to these choice ratios, then used to measure the certainty equivalents (CEs) of individual gambles (the safe magnitude for which the probability of either choice was 0.5; black arrow). The solid vertical line indicates the expected value (EV) of the gamble represented in the box. **d** Estimating the strength of preferences from riskless choices. Riskless safe rewards were presented against one another, the probability of choosing the higher magnitude option (A) is plotted on the y-axis as a function of the difference in magnitude between the two options presented ($\Delta$ magnitude). The differences in magnitude tested were 0.02 ml, 0.04 ml, 0.06 ml, and a psychometric curve, anchored with its inflection anchored at a $\Delta$ magnitude of 0, were fitted on the choice ratios measured (Eq. 2). These functions were fitted to different magnitude levels, and the temperature of each curve was linked to the strength of preferences at each of these different levels

Two male rhesus macaques (*Macaca mulatta*; Monkey A: 11.2 kg, Monkey B: 15.3 kg) participated in this experiment. The animals were born in captivity at the Centre for Macaques (CFM) in the UK and were pair-housed for most of the experiment. Monkey A (‘Tigger’) had been surgically implanted with a headpost under full anesthesia and aseptic procedures for subsequent neurophysiological recordings; he was not headposted for the current experiment. Monkey B (‘Ugo’) had been surgically implanted with a headpost and a recording chamber for neurophysiological recording; he was headposted for 2–3 h on each test day of the current experiment, which was intermingled with neuronal recordings on separate days. Both animals had previous experience with the visual stimuli and experimental setup (Ferrari-Toniolo et al. 2019). Each animal was seated in a primate chair (Crist instruments) in which he chose on each trial using a left–right moveable joystick (Biotronix Workshop, University of Cambridge) between two reward options (reward-predicting stimuli) presented on an upright computer monitor in front of them; he received the reward he had selected at the end of each of these binary choice trials (Fig. 1a).

### Task design and setup

The premise of this study was to compare the utility functions estimated from monkeys’ choices in risky or riskless decisions. To do so, monkeys were presented with sets of choices that could then be translated into utility metrics. The utilities derived from risky choices were compared with utilities measured from riskless choices, first assuming no subjective weighting of probabilities (EUT utilities), then accounting for the contribution of probability weighting (PT utilities). While these tests were specifically tailored to the requirement of elicitation of utility functions, they did not encompass the wide spectrum of daily risky choices.

Reward options took the form of various combinations of reward magnitude and probability and were represented on the monitor through horizontal lines that scaled, and moved, relative to two vertical ‘framing’ lines (Fig. 1b). Reward magnitudes were represented by the vertical position of the horizontal lines: 0 ml at the bottom of the vertical frame (1.5 ml at the top, and 0 < m < 1.5 in between), whilst the probability of receiving said reward was represented by the width of the horizontal lines within the frame. A single, horizontal line that touched the frames at both ends signaled a certain reward (probability *p* = 1); multiple lines that failed to touch the frames indicated gambles with probabilistic outcomes, each with associated probability 0 < *p* < 1 (Fig. 1a). The monkeys were habituated, trained and tested for associations between these two-dimensional visual stimuli and the blackcurrant juice rewards over the course of two years; both monkeys had previous experience with the task and stimuli before this study. Thus, while being different from behavior measured in holding cages or the wild, the experimental durations assured acceptable standards of reliable performance with well-controlled stimuli and rewards. Both animals had experienced reward probabilities that ranged from 0 to 1 (Ferrari-Toniolo et al. 2019), and reward magnitudes that ranged from 0 ml to 1.3 ml of juice. For this study, reward magnitudes were held between 0 ml and 0.5 ml of blackcurrant juice, and reward probabilities ranged from 0 to 1.

Each binary choice trial began with a white cross at the center of a black computer monitor, if the monkey were holding the joystick, a cursor would also appear on the monitor (Fig. 1a). Using the joystick, the monkeys initiated each trial by moving the cursor to the center cross and holding it there for 0.5–1 s. Following this holding period, two reward options appeared to the left and to the right of the central cross (see Fig. 1a). The animal had 3 s to convey his decision by moving the joystick to the selected side and holding his choice for 0.1–0.2 s—the unselected option would then disappear. The selected option lingered on the monitor for 1 s after reward delivery—followed by a variable inter-trial period of 1–2 s before the next trial. Errors were defined as unsuccessful central holds, side selection holds, or trials where no choices were made. Each of these resulted in a 6 s timeout for the animal, after which the trial would be repeated (ensuring the elicitation of preferences for each tested option set). Additionally, all reward options were repeated on both the left and right sides of the computer monitor, alternating pseudo-randomly to control for any side preference. Both the joystick position and task event times were sampled and stored at 1 kHz on a Windows 7 computer running custom MATLAB software (The MathWorks 2015a; Psychtoolbox version 3.0.11). We collected on average 423 ± 91 (SD) trials per session over 22 sessions for monkey A, and 338 ± 41 trials over 7 sessions for monkey B. Only one session was run on a given experimental day, and 3–5 sessions were run on each week. Thus, beyond the initial general habituation and task performance for similar tasks for another study mentioned above (Ferrari-Toniolo et al. 2019), the experiment lasted for several weeks with each animal after performance in the current task had been established. Only trials in which a given option set had been repeated at least four times were analyzed. Data processing and statistical analyses were run in Python (Python 3.7.3, SciPy 1.2.1).

The progression of testing with on-going reward consumption on a given day may result in satiety. We used several measures to control for potential confounding effects. First, specific satiety tests on other monkeys performing similar tasks in our laboratory had demonstrated that the currently used blackcurrant juice reward induced minor satiety compared to other juices (Pastor-Bernier et al. 2021). Second, we terminated testing for the day when task performance degraded rapidly, as indicated by increased errors in central hold, side selection and no choice; from that point on the animal would stop performing any task within 30–50 trials. Third, to directly compensate for potential effects on the utility function, we ran the risky and riskless choice sequences either on alternating days or started in alternation on a given day; thus, any potential satiety effect on the estimated utility function would not selectively affect only one type of utility function. Thus, while satiety may inadvertently affect any repeated tests of stochastic choice to some extent, the effects in our experiment would apply to both types of utility function and thus have minor effects on their comparison, which was the goal of the present experiment.

### Revealing preferences for risky and riskless choice

The monkeys’ daily reward preferences were measured in risky and riskless choice sequences under the framework of utility maximization. In risky choice sequences, trials always pit a risky gamble against a safe option—the utility of different reward magnitudes was estimated via the ratio of choices between different gamble and safe rewards. All gambles comprised two equally likely reward outcomes (though one could be 0 ml). In riskless choice sequences, monkeys were presented with two ‘safe’ options, each with a single fixed outcome—we used the ratio of choice between the two rewards to estimate utility. From these empirical data, we statistically estimated distinct utility functions from risky and riskless choices using, respectively, a PT-based discrete choice model (Tversky and Kahneman 1992) and the random utility maximization (RUM) model (McFadden 1974), as detailed below.

### Estimating utility functions in risky choice

**Fig. 2:**
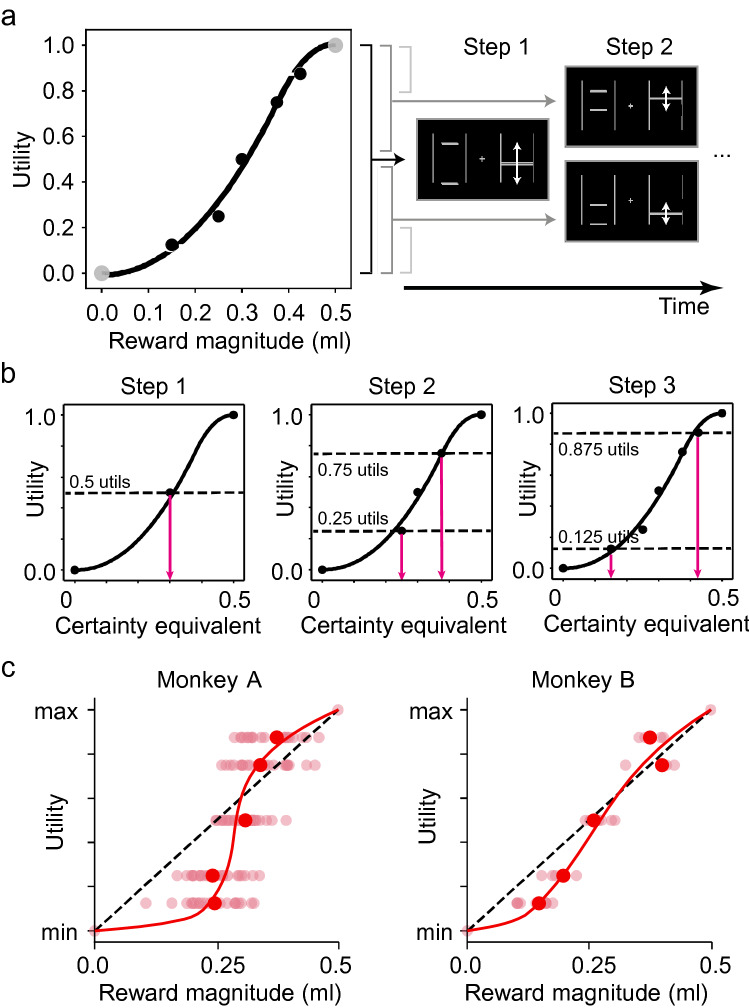
Estimating risky utilities using the fractile procedure. **a** Fixed utilities are mapped onto different reward magnitudes. The gambles that monkeys experienced are defined from bisections of the range of possible reward magnitudes. For each step the gambles were held fixed; safe magnitudes varied by 0.05 ml increments. **b** Estimation of utility using the stepwise, fractile method. In step 1, the monkeys were presented with an equivariant gamble comprised of the maximum and minimum magnitudes in the tested reward range. The CE of the gamble was estimated and assigned a utility of 50%. In step 2, two new equivariant gambles were defined from the CE elicited in step 1. The CEs of these gambles were elicited and assigned a utility of 25% and 75%. Two more gambles are defined in step 3, from the CEs elicited in step 2. Their CEs were then assigned a utility of 12.5% and 87.5%. Parametric utility functions, anchored at 0 and 1, were fitted on these utility estimates (see methods). **c** Utility functions estimated from choices. Data points represent daily CEs (semi-transparent) and their median values (red filled circles) tied to specific utility levels, as estimated through the fractile procedure. Both monkeys exhibit risk-seeking behaviour for low-magnitude rewards, and risk-aversion for high-magnitude ones. The data represent individual utility estimates gathered over 22 sessions for monkey A, and 7 sessions for monkey B. The red curves were obtained by fitting piecewise polynomial functions to the measured CEs (cubic splines with three knots)

For risky sequences, utilities were estimated using the fractile-bisection procedure—a method that involves dividing the range of possible utilities into progressively smaller ranges and estimating the reward magnitude associated with each of these utility ranges (Fig. 2). The animal chose between a gamble that was set to two specific and equiprobable magnitudes (between 0 and 0.5 ml; *p* = 0.5 each) and a safe reward whose magnitude was varied across the whole range of 0 ml to 0.5 ml. We psychometrically estimated the choice indifference point at which each option was chosen with equal, 0.5 probability and determined the ‘certainty equivalent’ (CE) as the magnitude of safe reward (in ml) that was subjectively equivalent to the utility of the gamble. Thus, depending on the animal’s risk-seeking or -avoiding attitude in a given part of the reward distribution, the CE, and thus the safe reward, could be, respectively, lower or higher compared to the mean gamble reward (i.e. the gamble’s Expected Value, EV, defined as summed products of reward magnitude and probability). The procedure defined set utility metrics (in this case ½, ¼ and ¾, and 1/8 and 7/8 of the maximum utility, see Fig. 2a, b) for which the equivalent safe rewards were derived (Fig. 2a).

To estimate the CE, we fitted the following logistic function to the proportion of safe choices for each gamble/safe option set:

1 $$P\left( {{\text{ChooseSafe}}} \right){ } = 1/(1 + { }e^{{ - \left( {\frac{{{\text{SafeReward}}_{ml} { } - { }x_{0} }}{\sigma }} \right)}} { ,}$$

where the probability that the monkeys would choose a safe reward over the gamble [P(ChooseSafe)] was contingent on the safe option’s magnitude ($${SafeReward}_{ml})$$ and two free parameters: *x*_0_, the x-axis position of the curve’s inflection point, and *σ*, the function’s temperature. Importantly, this function’s inflection point represented the exact safe magnitude for which the monkeys should be indifferent between the set gamble and a given safe reward. The *x*_0_ parameter could thus be used as a direct estimate of the gamble’s CE or, put simply, the safe reward equivalent to the utility level being tested. Only sequences that contained a minimum of three different option sets (repeated at least 4 times) were used in the elicitation of CEs.

From the first CE, identified as having 0.5 utility, two new equiprobable gambles were created representing utility values of 0.25 (¼ of the utility range) and 0.75 (1/4 and ¾ of the utility range, respectively). Of the two new gambles, one was set between 0 ml and the first CE’s ml value, and the other was set between the first CE and 0.5 ml (Fig. 2b). The CE elicitation procedure (logistic fitting, Fig. 1c) was repeated for each of these gambles. All option sets were interwoven in the same sequence to ensure a similar spread in the presented rewards. Further details of this procedure are described in Supplementary Information.

### Estimating utility functions in riskless choice

For riskless choice sequences, choice ratios between two safe options were measured as likelihood of choosing the high-magnitude option over the lower-magnitude one (Fig. 1d). The range of juice rewards (0.05–0.5 ml) was divided into 0.05 ml increments and the options were centered on these magnitude increments. For each increment, we defined three sets of options, using reward magnitudes differences of 0.02 ml, 0.04 ml and 0.06 ml, which are hereafter called ‘gaps’. The small size of these differences ensured that choices would be stochastic. Each gap was anchored at its respective ‘midpoint’.

**Fig. 3:**
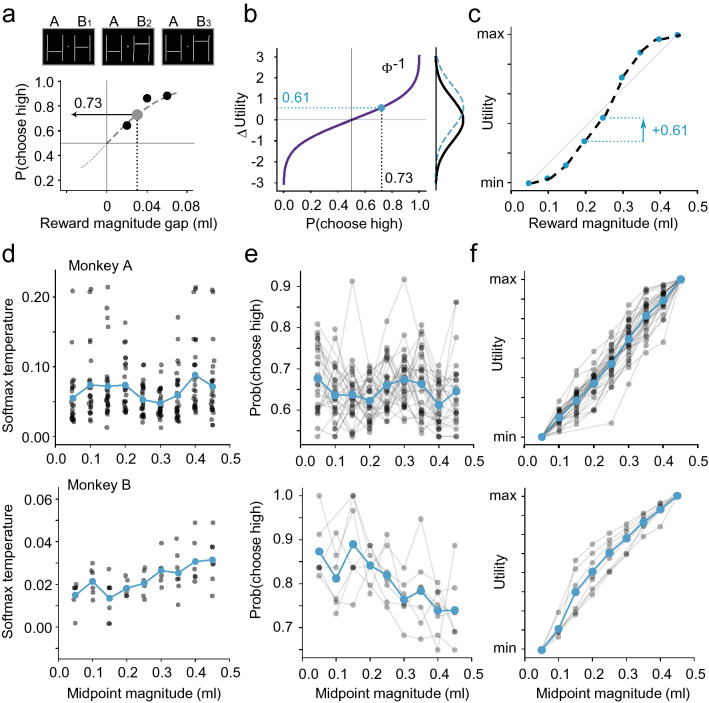
Estimating riskless utilities from the stochasticity in safe–safe choices. **a** Measuring stochasticity in choices between safe two reward options. Example visual stimuli (top) representing choices between safe rewards (A: low, B: high) resulting in different percentage of choices for the high option (bottom; black dots). This was repeated for different reward option sets, centered at different increments (midpoints). For each midpoint, the likelihoods were fitted with a softmax curve (dashed), used to estimate the probability of choosing the larger option for a gap of 0.03 ml (gray dot). **b** Choice ratios as differences in utility. The likelihoods that monkeys would pick the better reward were transformed using the inverse cumulative distribution function (iCDF) of a logistic distribution. The utility of different rewards took the form of equally noisy distributions centered at the true utilities. The output of iCDFs is the distance between these random utilities (i.e. the marginal utility). **c** From marginal utilities to utility. The cumulative sum of marginal utilities approximated a direct utility measure for each midpoint. These measurements were normalized whereby the utility of the highest midpoint was 1, and the starting midpoint had a utility of 0. **d** Daily strength of preference estimates. Each point represented the temperature of the softmax curve fitted on the choice ratios (blue points: average across days). The lower the temperature parameter, the steeper was the softmax curve and the more separable were the random utilities. Lower values meant higher marginal utility measurement (steeper utility function), higher ones meant lower marginal utility (flatter function). **e** Daily choice ratio estimates from softmax fits. Estimates from the same day are linked by grey lines. Ratios of 0.5 meant that the random utility of the two options were fully overlapping (i.e. flat utility function); choice ratios closer to 1 meant random utilities that were fully dissociated and non-overlapping. **f** Utility functions. Utilities estimated in single days (grey lines) and averages (blue), normalized relative to the minimum and maximum midpoint

The likelihood of choosing the higher magnitude option in different gap-midpoint option sets was used to infer the shape of the utility function (Fig. 3a, b, c). Specifically, the difference between the likelihoods of choosing the better options, at different midpoints, reflected the separability of the utility of different reward magnitudes. Under RUM, the degree of certainty with which choices are made (i.e. the closer choice ratios are to 100%) directly correlates with the separability of the noisy utilities that correspond to each option in a choice. This implies that, looking at repeated choices between two set magnitudes, a decision-maker with a flatter utility function should exhibit more stochasticity in their choices (i.e. less precision) than a decision-maker with a steeper utility (i.e. more precision). Changes in choice ratios between sequential midpoints, as averaged across gaps, could therefore be used as a proxy for the slope of the utility function.

To estimate these RUM-compliant utilities, logistic curves were fitted to the likelihood of choosing the better option (for the three gaps) at every midpoint level (Fig. 3a):

2 $$P\left( {{\text{ChooseHigher}}} \right){ } = 1/\left( {1 + { }e^{{ - \left( {\frac{{{\text{Gap}}_{ml} { }}}{\sigma }} \right)}} } \right){ }{\text{.}}$$

Unlike for CE estimation, this logistic function captured the likelihood of choosing the high-magnitude option (in a safe-safe option set) contingent on the gap between the two options (${\mathrm{Gap}}_{ml}$) and *σ*, the logistic function’s ‘temperature’ parameter. Further analyses of the probability that the monkeys would pick the better reward (${x}_{i}$) employed the following equations:

3 $$P\left( {x_{i} } \right){ } = { }P\left[ {U\left( {x_{i} } \right){ } \ge { }U\left( {x_{j} } \right)} \right],$$

4 $$P\left( {x_{i} } \right){ } = { }P\left[ {u\left( {x_{i} } \right){ } + { }\varepsilon_{i} { } \ge { }u\left( {x_{j} } \right){ } + { }\varepsilon_{j} { }} \right],$$

5 $$P\left( {x_{i} } \right){ } = { }P\left[ {u\left( {x_{i} } \right){ } - { }u\left( {x_{j} } \right){ } \ge { }\varepsilon_{j} { } - { }\varepsilon_{i} { }} \right],$$

In this form, the probability of choosing ${x}_{i}$ rather than ${x}_{j}$ was given by the probability that the difference in the true utilities of ${x}_{i}$ and ${x}_{j}$ was greater or equal to the noise on ${x}_{j}$ (${\varepsilon }_{j}$) minus the noise on ${x}_{i}$ (${\varepsilon }_{i}$). From this, it followed that the distribution of noise differences could be used as a predictor of the distance between the two true utilities [$u({x}_{i})$ and $u({x}_{j})$]. Because of the assumption of constant noise, the probability of choosing ${x}_{i}$ over ${x}_{j}$ would be directly proportional to the distance between the true utility of two options. In accordance with McFadden’s formulation (McFadden 1974, 2005; Stott 2006), we assumed that the distribution of error differences (${\varepsilon }_{j} - {\varepsilon }_{i}$) took a logistic form:

6 $$P\left( {x_{i} } \right){ } = { }\frac{1}{{\left( {1 + e^{{ - \Delta utility{ }}} } \right)}}{ ,}$$

And then used the inverse of this logistic distribution’s CDF to estimate the difference in utilities ($\Delta \mathrm{utility}$) between hypothetical 0.03 ml reward gaps (Fig. 3b)—essentially the slope of the utility function at every midpoint (Fig. 3c). The cumulative sum of these slopes provided an estimate of the utility at each midpoint. Further details of this procedure are described in Supplementary Information.

### Estimating utility functions from risky and riskless choices in a common metric

To directly compare the utility functions between risky and riskless choices, we re-estimated utilities on a common scale, compatible with PT. We used the same discrete choice model to describe both risky and riskless choices, without the need of two different estimation procedures.

The main assumption of our model is that a random quantity is added to each option’s utility at every trial, using the PT model as the underlying deterministic choice mechanism. This model introduced stochasticity in choices and could readily be applied to both risky and riskless choices without modification.

In the model, utility functions took the form of the cumulative distribution function of a two-sided power distribution (Eq. 10; Kotz and Dorp 2010), a 2-parameter function that could easily account for complex risk attitudes (Kontek and Lewandowski 2018): if $\alpha$ < 1, the utility function would be convex and predict risk-seeking choices up to the inflection at parameter $\kappa$ (predicting risk-averse choices thereafter); if instead $\alpha$ > 1, the utility function would be concave and predict risk-averse behavior up to the inflection at $\kappa$ (predicting risk-seeking behavior afterwards). For risky choices, a 1-parameter power function captured the weighting of probabilities (Eq. 9). Since the only probability experienced was *p* = 0.5, $\rho$ < 1 implied an overweighting of the probability of receiving the highest reward whilst $\rho$ > 1 implied underweighting.

We defined three forms of this discrete choice model, with different free parameters: the EV model (linear utility and probability weighting), where only the “noise” parameter was free to vary; the EUT model (linear probability weighting), where the utility parameters could vary; and the PT model, with both utility and probability weighting free parameters. In risky choices, we compared the goodness-of-fit of the three models to identify the one that would produce the best estimate of a utility function. In riskless choices, we estimated the utility function using the EUT model.

This analysis placed utility metrics for risky and riskless choices on a common and comparable scale, and, importantly, it allowed for the inclusion of probability weighting as an additional contributor to the monkey’s preferences. As in most discrete choice models (and in line with the aggregate RUM metric), a logit function (softmax) was used to represent noise in the decision-making process. Details of this procedure are described in Supplementary Information.

### Statistical comparison of risky and riskless choices

Estimating utilities through discrete choice modelling allowed for the comparison of the functional parameters that best described decisions in risky and riskless choices, and to explore the unique contributions of both magnitudes (through utility) and probabilities (through probability weighting) in a way that aggregate, non-parametric measures did not permit.

Because the logit function’s $\lambda$-, and the utility’s *α* parameters were asymmetrically distributed (with positive values < 1 accounting for as much change as values > 1), these were log-transformed before proceeding with any comparison. Then, the parameters elicited in risky choice sequences were compared to those estimated from riskless sequences using a one-way multivariate analysis of variance (or MANOVA) whereby the main comparison factor in the analysis was the risk–riskless choice scenario described by each set of parameters. Since the probability weighting parameter for riskless choices was constant and fixed at 1, we restricted the MANOVA analysis to the softmax and utility parameters. We then ran additional correlation analyses (Pearson’s R) between risky and riskless utility parameters to determine if the parameters in one set of choices could predict those of another.

As utility represents the subjective preferences of individual monkeys, all parameters were compared independently for each monkey, results were never pooled across animals, and the statistics for each monkey are reported separately. Thus, the results are considered generalizable with limits given by the variations between individual animals. All statistical analyses were considered significant at *p* < 0.05.

## Results

### Experimental design

Each animal chose between two options presented on the left and right halves of a computer monitor by moving a joystick towards the chosen side (Fig. 1a). The reward options varied in terms of blackcurrant juice quantity as well as in the probability that they would be delivered. The monkeys received the selected rewards after every trial—contingent on their delivery probability. Choice preferences were elicited in trial sequences in which either both options were certain and therefore riskless, or in sequences in which one option was certain (safe option) and the other was a risky gamble with two possible outcomes (juice magnitudes), each delivered with probability *p* = 0.5 (equiprobable gamble). We separately used these riskless or risky choices to infer an animal’s utility function.

### Utility functions in risky and riskless choice

Choices were measured during 22 and 7 days with monkeys A and B, respectively. On each of these days, the animals were tested in both risky and riskless choices, using the certainty equivalent (CE) with the fractile procedure (Fig. 2) and the random utility maximization (RUM) procedure (Fig. 3), respectively. For both risky and riskless sequences, a link between utility measurements and reward magnitudes was confirmed via one-way ANOVA. Both monkeys exhibited a significant main effect of utility on the CEs (Fig. 2c) in risky choices (Monkey A: F_(4,124)_ = 35.482, *p* = 9.763∙10^–20^, Monkey B: F_(4,39)_ = 172.537, *p* = 3.090∙10^–24^). In riskless choices, we contrasted the utilities with the midpoint reward magnitude (Fig. 3f), highlighting a significant main effect (Monkey A: F_(8,232)_ = 375.763, *p* = 3.503∙10^–128^; Monkey B: F_(8,52)_ = 85.561, *p* = 3.474∙10^–27^). These basic results illustrated how the utilities associated with different reward magnitudes were significantly different from each other, which would not have been the case if monkeys selected options at random.

Importantly, the utility levels were significantly rank-ordered in relation to the reward magnitudes (Spearman rank correlation in Monkey A: risky Rho = 0.7209, *p* = 5.853∙10^–22^; riskless Rho = 0.9628, *p* = 8.035∙10^–138^. In Monkey B: risky Rho = 0.9446, *p* = 6.092∙10^–22^; riskless Rho = 0.9665, *p* = 1.529∙10^–36^), in line with the fundamental principle of utility functions being monotonically related to the reward magnitudes. In general, utilities appeared to be non-linear functions of physical reward magnitudes.

In risky choices, the full-elicited risky utility functions followed an S-shaped pattern in both monkeys, reflecting the typical risk attitudes observed in macaques: risk-seeking (convex utility) for relatively low-magnitude rewards and risk-aversion (concave utility) for relatively high-magnitude ones (Fig. 2c).

In riskless choices, we compared the estimated utility increments to highlight any non-linearity in the utility shape. As increments in utility were proportional to the temperature parameter (i.e. the slope) of the softmax curves that described choices around a certain magnitude level, the softmax temperature could be used as a proxy for linearity: a constant temperature across magnitude levels would correspond to a linear utility function, while a varying temperature would indicate non-linear utility. We compared the temperature parameter across midpoints and found that it varied significantly with magnitudes (Fig. 3d; Monkey A: F_(8, 232)_ = 2.663, *p* = 8.165∙10^–3^); Monkey B: F_(8, 52)_ = 4.187, *p* = 6.370∙10^–4^) highlighting the non-linearity in the riskless utility function, in both monkeys. The softmax temperature, as a function of the midpoint, reached a minimum (around 0.30 and 0.15 ml for monkeys A and B, respectively) before increasing again, suggesting a slight S shape for the riskless utility function (Fig. 3f).

Although these aggregate utility measures were based on commonly defined economic models, they were not (i) PT-compatible, and (ii) comparable between the risky/riskless choice scenarios. In fact, we estimated the risky utility functions following EUT, which, in contrast with PT, assumes no subjective weighting of probabilities; the utility functions had different magnitude ranges in risky and riskless choices (0–0.5 ml and 0.05–0.45 ml, respectively) and different discrete steps. We sought to overcome these limitations by defining a utility estimation method that allowed for a direct comparison of utility in risky and riskless choices, compatibly with economic choice models.

### Risky and riskless utility functions on a common scale

**Fig. 4:**
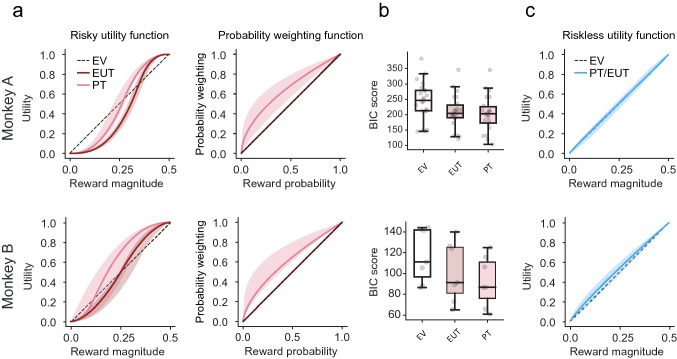
Discrete choice estimates differ between risky and riskless choices. **a** Utility functions in risky choice. Median parametric estimates for utility functions and probability weighting functions fitted to risky choices. Shaded area: 95% C.I. on the median of these functions. Two versions of the discrete choice model were fitted: the expected utility theory (EUT) model predicted choices solely based on reward options’ utilities (without probability weighting); the prospect theory (PT) model, predicted choices based on utilities and probability weighting. An expected value (EV) based model was included for comparison. Monkeys were risk-seeking, but where the PT model accounted for this mainly through probability weighting, the EUT model accounted for it through a more convex utility. **b** Comparison of risky choice models. The PT model described individual choices better than EUT and EV. Bayesian information criterions (BIC) were calculated from the log likelihoods of the daily best-fitting PT and EUT discrete choice models. **c** Utility functions in riskless choice. Median parametric estimates for utility functions fitted to riskless choices (shaded area: 95% C.I. on the median). The discrete choice model predicted choices from the expected utilities of rewards (no probability weighting). Utilities were mostly linear, though slightly concave

In risky choices, both the EUT and PT models predicted S-shaped utility functions (Monkey A EUT: *t*_(22)_ = − 29.0190, *p* < 0.00001; Monkey A PT: *t*_(22)_ = − 28.2543, *p* < 0.00001; Monkey B EUT: *t*_(22)_ = − 4.2859, *p* = 0.005172; Monkey B PT: *t*_(7)_ = − 7.4532, *p* = 0.000301) (Fig. 4a, b). The PT model, however, relied on concave probability weighting (one-sample *t* test, Monkey A: *t*_(22)_ = − 4.2533, *p* = 3.55 × 10 × ^−4^; Monkey B: *t*_(7)_ = − 2.7316, *p* = 0.0341), rather than a convex utility function, to explain risk-seeking behavior. For that reason, PT’s S-shaped utility functions were mostly left-skewed (more concave than convex), whereas EUT utility functions captured risk-seeking behavior solely through a right-skewed S shape (more convex than concave) (Fig. 4a). Overall, the daily best-fitting parameters from the PT and EUT models were significantly different from each other (Table 1), with the PT model capturing behavior significantly more reliably than both EV and EUT models (Fig. 4b; Wilcoxon rank-sum test; monkey A: *p* = 1.0  ×  10^–4^ $;$ monkey B: *p* = 1.8 × 10^–2^). Through the PT model, we could separate the contribution of utility and probability weighting to the risk attitude, obtaining a better estimate of the utility function underlying choices, compared to the EUT model.

**Table 1:** MANOVA tests for pairwise differences between the risky EUT, risky PT, and riskless discrete choice models

	Utility type	F (1, 42)	*p*	Wilks *λ*
Monkey A	Riskless, risky (PT)	28.697	6.158 × 10^–12^	0.209
	Risky (EUT), risky (PT)	5.475	6.856 × 10^–4^	0.581

	Utility type	F (1, 12)	*p*	Wilks *λ*
Monkey B	Riskless, risky (PT)	8.744	4.239 × 10^–3^	0.155
	Risky (EUT), risky (PT)	1.687	0.243	0.487

The analyses were run on four of the five free parameters, excluding probability weighting. The risky EUT and riskless models had no probability weighting parameter to compare with the risky PT model’s probability weighting

In riskless choices (Fig. 4c), the utility function’s *α* parameter was not significantly different from one (*t* test, Monkey A: *t*_(22)_ = − 0.3267, *p* = 0.7471; Monkey B: *t*_(7)_ = 1.3457, *p* = 0.2270). This implied that the riskless utility functions were close to linear, suggesting that magnitudes were objectively represented, according to the RUM framework.

### Mismatch between risky and riskless utility functions

**Fig. 5:**
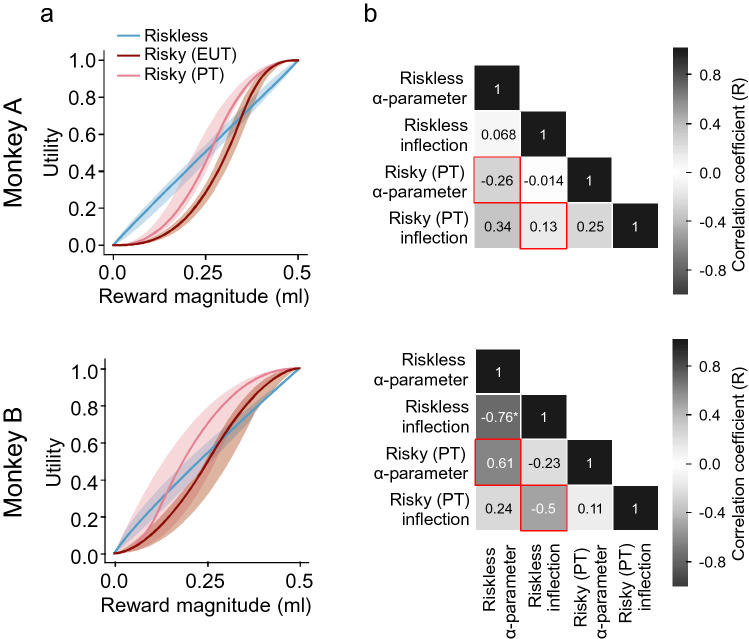
Risky utilities do not predict riskless ones, and vice versa. **a** Median utility function estimates for risky and riskless choices. The shaded area represents the 95% C.I. on the median of these functions. For riskless choices, utility estimates were mostly linear (though slightly concave). For risky utilities, the two different versions of the discrete choice model predicted S-shaped utilities, but risky EUT utility functions were more convex than PT utility functions. **b** Absence of correlation for utility parameters in risky vs. riskless choices. Pearson’s correlations were run on the parameters from risky and riskless scenarios. Red squares highlight Pearson’s R for the correlation of the α and inflection parameters between risky and riskless choices. Asterisks (*) indicate significant correlations (p < 0.05)

We compared the shapes of risky and riskless utilities computed on a common scale, in terms of the two utility parameters, *α* and $\kappa$. The *α* parameter represented the non-linearity of the utility function: S-shaped (*α* < 1), inverse-S-shaped (*α* > 1) or linear (*α* = 1); the $\kappa$ parameter represented the inflection point where the curvature of the utility function would invert. We found a significant difference in the utility functions’ shapes in terms of the α parameter, in both monkeys (Fig. 5a; Monkey A: F_(1,42)_ = 72.717, *p* = 1.04 × 10^–10^; Monkey B: F_(1,12)_ = 24.221, *p* = 3.52 × 10^–4^).

Monkey B’s difference in the utility’s inflection point between risky and riskless choices (Monkey A: F_(1,42)_ = 1.282, *p* = 0.264; Monkey B: F_(1,12)_ = 17.153, *p* = 0.00136) was significant, while we found no significant difference in either the noise or the side bias parameters (noise: Monkey A: F_(1,42)_ = 2.760, *p* = 0.104; Monkey B: F_(1,12)_ = 0.182, *p* = 0.677; side bias: Monkey A: F_(1,42)_ = 0.2407, *p* = 0.626; Monkey B: F_(1,12)_ = 2.338, *p* = 0.152).

Overall, these results show that the dissimilarity between the modeled risky and riskless choices was mainly due to a difference in the non-linearity of the utility functions, as expressed by the *α* parameter. The utility function was strongly non-linear in risky choices, while it was close to linear in riskless choices.

The difference in utility functions was also evident when comparing risky and riskless data from single days, through a correlation analysis: we found was no significant correlation between any of the parameters of risky utility functions and those of riskless utility functions across days (Fig. 5b).

As a control, we correlated the measured riskless choice percentages (for the hypothetical 0.03 ml gap, grey dot in Fig. 3a) with the modeled ones, separately using the utility function elicited from risky or riskless choices. We found significant correlation coefficients when predicting riskless choices using the riskless utility function (Monkey A: R = 0.442, *p* = 1.278 × 10^–9^; Monkey B: *R* = 0.484, *p* = 7.610 × 10^–5^) but not using the risky one (Monkey A: *R* = 0.104, *p* = 0.175; Monkey B: *R* = 0.087, *p* = 0.503). Thus, the riskless utility function captured the behavior in riskless choices while the risky utility function did not, emphasizing the difference in risky/riskless utilities.

In summary, estimating utilities by including the subjective probability weighting of PT, rather than EUT, brought risky fits more in line with riskless ones (Table. 1; Fig. 5a) in line with previous human studies (Stalmeier and Bezembinder 1999; Abdellaoui et al. 2007). However, a direct comparison between the risky and riskless utility parameters revealed significant differences in the utility functions’ shapes between the two choices scenarios (Fig. 5b).

## Discussion

Using a robust, incentive-compatible task, we showed that utility functions that describe decisions involving risk more closely mimicked riskless utility functions when probability weighting was considered. To compare utility functions between categorically distinct risky and riskless scenarios, we derived these functions statistically from empirical data of macaque monkeys’ choices, using stochastic versions of PT and EUT. These analyses resulted in reliably estimated functional parameters that best described their empirically assessed economic choices. Each day, the monkeys were presented with risky or riskless binary choice sequences. In risky ones, they made choices between gambles and safe rewards; in riskless ones, both choices had a single, certain outcome. We found that analyzing monkeys’ risky choices by including the subjective probability weighting of PT, in addition to providing a better fit than EUT, led to decision parameters that more closely resembled those of riskless choice. This trend is in line with the human literature (Stalmeier and Bezembinder 1999; Abdellaoui et al. 2007). However, the comparisons between utility functions differed: the monkeys’ utility functions elicited in risky and riskless choices were more alike after including the subjective weighting of probabilities, while still differing significantly. 

The CEs estimated in fractile sequences of risky choices suggested that both monkeys were risk-seeking for all but the highest reward magnitudes. The risk-seeking attitude was robust irrespective of consideration of probability weighting (PT model) or its neglect (EUT model). However, the way the two models accounted for risk-seeking differed. The PT model captured risk-seeking by concave probability weighting (the subjective probability of winning a gamble was higher than the objective probability). By contrast, the EUT model captured risk-seeking exclusively by convex utility. Although the inclusion of concave probability weighting in the PT model indicated somewhat less risk-seeking behavior than the EUT model would predict, both models were consistent in suggesting risk-seeking behavior. By contrast, the riskless choices might suggest a somewhat different decision mechanism. Here, the relatively linear (if slightly concave) riskless utility function suggested closer influence of objective reward magnitude and thus less subjectivity in choices. It appears that, at least within the confines of our experiment, the cognitive processes underlying economic decisions might differ between risky and riskless choices in a way that is beyond the simple addition of probability weighting.

While the same binary choice design was used in risky and riskless choices, the difference between options was much greater in risky sequences than in riskless ones. To estimate aggregate riskless utilities, for example, the rewards that the monkeys experienced differed only by up to 0.06 ml in every trial. In risky sequences, on the other hand, gambles were pitted against safe rewards spread over the full range of the gambles’ outcomes. Monkeys experienced a broad range of magnitudes in each of the sequences, but the differences between riskless choices could have required far more attention to dissociate than those in riskless choices (something we cannot account for; but see, Farashahi et al. 2018). Thus, larger variations of risky options that respect the wide spectrum of risk preferences could be used for more complete risk tests and might provide helpful information for refining current decision models.

As a limitation of the current study, the discrepancy between risky and riskless utility functions could be influenced by our model specifications. Our study employed some of the more widely used and well tested fitting models. However, such models could result in an oversimplification, and alternative models should be compared to support our conclusions. In particular, we assumed a constant and symmetric noise around each option’s utility, whereas a more biologically plausible contribution of noise on the utility measure could include asymmetric and non-constant noise (especially for activity rates close to the limits of the neurons’ dynamic range). Also, different noise distributions could be applied to the option components (magnitude and probability). Thus, follow-up studies with more refined models might include different assumptions on the noise shape.

Where these findings fail to replicate the data from risky and riskless introspective studies (though see Hertwig et al. 2018), they are nonetheless in line with the incentive-compatible time trade-off approach. As these types of time discounting tasks are easily adapted to study preferences in rhesus macaques (Hayden and Platt 2007; Kobayashi and Schultz 2008; Hwang et al. 2009; Blanchard et al. 2013), it would be interesting to see how utility functions estimated using time trade-offs in macaque monkeys correlate with the present findings. Another approach could be to compare risky and riskless choices between bundles of outcomes; here choice indifference arises from the combinations of rewards. Previous such studies demonstrated variations between risky and riskless choices when some outcomes involved losses (Chung et al. 2019). Monkeys show win-stay–lose-shift strategies (Gilovich et al. 1985; Barron and Erev 2003; Heilbronner and Hayden 2013) and reverse risk preferences for gambles depending on previously wins or losses (Lau and Glimcher 2005; Blanchard et al. 2014; Ferrari-Toniolo et al. 2019). Thus, future studies might estimate utility functions from trial-by-trial changes rather than globally from an entire experimental procedure.

As typical for scientific studies, our results have generality constraints (Simons et al. 2017). First, we used only two monkeys, primarily for reasons of ethics toward a species close to humans. We did not pool the data from the two animals as reward functions in general and utility estimates in particular are subjective and thus should not be averaged across subjects. Even without pooling, two rhesus monkeys may not be representative for the whole species, and other individuals of the same species may show some behavioral differences. This limitation can be partly overcome by comparing data across different laboratories. However, despite expected individual variations, the data from the two animals were reproducible, which we deemed as most important aspect. Second, monkeys are not humans, nor rats, and the obtained data should be compared to those from other species. Third, we used specific tasks in a laboratory setting. While tasks and laboratory are typical for well-controlled empirical work, generality can only be achieved by comparing these data with those from other, equally well-controlled tasks. The laboratory situation differs notably from natural, free ranging behavior, and the intuition behind such a traditional approach is that the observed phenomena would underlie any natural behavior, which again requires comparisons. Fourth, our analysis of measured, empirical data applied current statistical methods to current economic models, including PT and RUM. As both economic models are less than 50 years old, whereas monkeys seek rewards for millions of years, future statistics and economic models may reveal additional, but hopefully not opposite, explanations of economic choice.

Assuming that the discrete choice model is correct, the difference in utility functions for risky and riskless utilities could be used as a quantitative basis for neuronal tests of utility coding. Monkeys could be using different strategies for solving the risky and riskless choice problems, implying different brain mechanisms. In particular, riskless choices are somewhat similar to perceptual discrimination in which subjective values derive primarily from individual needs and optimal solutions require perceptual comparisons of predictive stimuli. By comparison, risky choices require in addition assessment of probability and computation of risk, both of which involve memory or inferential processes depending on the frequentist or Bayesian approach to probability. Given these differences between risky and riskless choices, utility coding may occur in neurons in different brain regions, and their pattern should reflect the different utility shapes elicited in the two choice scenarios.

Overall, the results presented here add to the need for decision models to account for flexible, context-specific preferences (Hayden et al. 2008; Heilbronner and Hayden 2016; Farashahi et al. 2018). For decision-theory as a whole, reconciling dynamic preferences with more traditional economic models would go a long way to making more accurate, descriptive predictions. While there are undoubtedly common cognitive processes underlying the various forms of economic decisions, there are differences in subjectively valuing rewarding outcomes, as evidenced by the observed differences in formal utility functions. The current study pointed to differences in cognitive processes between riskless and risky decisions. It will be interesting to see whether similar utility differences, indicative of different underlying cognitive processes, can be identified with other variations of economic choice, such as time constraints, time delays and natural vs. artificial outcomes. Monkeys are particularly suitable for such investigations due to their capacity to perform thousands of trials, which allows researchers to test the same agent in different choice scenarios and acquire sufficient data for thorough model comparisons.

## Supplementary Information

Below is the link to the electronic supplementary material.

Supplementary file1 (DOCX 47 KB)
