# Supplementary material for: Comparing utility functions between risky and riskless choice in rhesus monkeys
Source: Anim Cogn. 2021 Sep 27;25(2):385–99. doi: 10.1007/s10071-021-01560-x (PMC8940808; doi:10.1007/s10071-021-01560-x)
Supplement: Supplementary file 1 — Supplementary file1 (DOCX 47 KB) [file 10071_2021_1560_MOESM1_ESM.docx]

**SUPPLEMENTARY INFORMATION**

Title: Comparing utility functions between risky and riskless choice in two rhesus monkeys.

Journal: Animal Cognition

Authors: Bujold, P.M, Seak, L.C.U., Schultz, W., Ferrari-Toniolo, S.

Affiliation: Department of Physiology, Development and Neuroscience, University of Cambridge, Cambridge CB2 3DY, United Kingdom.

E-mail addresses: Wolfram.Schultz@Protonmail.com, Simone.FerrariToniolo@gmail.com

**SUPPLEMENTARY METHODS**

For reasons of completeness and readability, some text in this section is replicated from the main text.

### Estimating utility functions in risky choice

Utility values of 0 and 1 were arbitrarily assigned to 0 ml and 0.5 ml of juice, respectively. Since monkeys only experienced trials set between these reward magnitudes, this constrained all utility estimates between 0 and 1. Then, in accordance with EUT, the utility of 0.5 was assigned to the equiprobable gamble formed of these two magnitudes. The first step of the procedure involved presenting the monkeys with choices between this gamble and varying safe rewards (in 0.05 ml increments), from these, the safe reward that was equivalent to the gamble in utility terms was identified (i.e. the safe reward chosen in equal proportion to the gamble; see Fig. 1c).

To estimate this safe reward, the following logistic sigmoid curve was fitted to the proportion of safe choices for each gamble/safe option set:

$P(ChooseSafe) =1/(1+ e^{-\left( \frac{{SafeReward}_{ml} - x_{0}}{\sigma} \right)}$ Eq. 1

where probability that the monkeys would choose a safe reward over the 0.5 utility gamble (P(*ChooseSafe*)) was contingent on the safe option’s magnitude (${SafeReward}_{ml})$and two free parameters: x_0_, the x-axis position of the curve’s inflection point, and σ, the function’s temperature. Importantly, this function’s inflection point represented the exact safe magnitude for which the monkeys should be indifferent between the set gamble and a given safe reward. The x_0_-parameter could thus be used as a direct estimate of the gamble’s CE, or, put simply, the safe reward equivalent to a utility of 0.5. Only sequences that contained a minimum of three different option sets (repeated at least 4 times) were used in the elicitation of CEs.

From the CE identified as the 0.5 utility value, two new equiprobable gambles were created representing utility values of 0.25 (¼ of the utility range) and 0.75 (1/4 and ¾ of the utility range, respectively). Of the two new gambles, one was set between 0 ml and the first CE’s ml value, the other was set between the first CE and 0.5 ml (Fig. 2b). The CE elicitation procedure (logistic fitting, Fig. 1c) was repeated for each of these gambles. Crucially, gamble/safe option sets for both gambles were interwoven in the same sequence – to ensure a similar spread in the presented rewards.

### Estimating utility functions in riskless choice

To estimate utilities compliant with random utility maximization (RUM), logistic curves were fitted to the likelihood of choosing the better option (for the three gaps) at every midpoint level (Fig. 3a):

$P(ChooseHigher) =1/(1+ e^{-\left( \frac{{Gap}_{ml}}{\sigma} \right)})$ Eq. 2

Unlike for CE estimation, this logistic function captured the likelihood of choosing the high-magnitude option (in a safe-safe option set) contingent on the gap between the two options (${Gap}_{ml}$) and σ, the logistic function’s temperature. Just as is the case for CE estimation however, the utility estimates relied on aggregate choices between multiple reward option sets. The logistic fit also highlighted sequences where monkeys would not follow even the most basic principle of rational choice: weak stochastic dominance (picking an objectively lower outcome). Choices where this was the case were removed from all future analyses: that is, when the estimated temperature parameters of logistic fits were negative (i.e. the larger the gap, the lower the likelihood of choosing the better option) or significant outlier (p < 0.05; Grubbs's test). In monkey A, 38 choice sets were removed from a total of 279 choice sets (14 negative parameters and 24 outliers). In monkey B, 1 choice set was removed from a total of 62 choice sets (1 negative parameters and no outliers).

Where logistic fittings were successful, the functions were used to estimate the higher-lower choice ratio, at each midpoint, for an untested magnitude gap of 0.03 ml (Fig. 3a). Then, the inverse cumulative of a logistic probability density function (centered at 0 with variance = 1) was used to estimate the distance, in utility terms, between the two magnitudes in the 0.03 ml gap (Fig. 3b). In other words, these 0.03 ml gaps were placed onto a shared scale (i.e. random utilities) through the assumption that, on each trial, the probability that the monkeys would pick the better reward ($x_{i}$) was given by:

$P(x_{i}) = P[U(x_{i}) \geq U(x_{j})],$ Eq. 3

$P(x_{i}) = P[u(x_{i}) + \varepsilon_{i} \geq u(x_{j}) + \varepsilon_{j} ],$ Eq. 4

$P(x_{i}) = P[u(x_{i}) - u(x_{j}) \geq\varepsilon_{j} - \varepsilon_{i} ],$ Eq. 5

In this form, the probability of choosing $\boldsymbol{x}_{\boldsymbol{i}}$ rather than $\boldsymbol{x}_{\boldsymbol{j}}$ was given by the probability that the difference in the true utilities of $\boldsymbol{x}_{\boldsymbol{i}}$ and $\boldsymbol{x}_{\boldsymbol{j}}$ was greater or equal to the noise on $\boldsymbol{x}_{\boldsymbol{j}}$ ($\boldsymbol{\varepsilon}_{\boldsymbol{j}}$) minus the noise on $\boldsymbol{x}_{\boldsymbol{i}}$ ($\boldsymbol{\varepsilon}_{\boldsymbol{i}}$). From this, it followed that the distribution of noise differences could be used as a predictor of the distance between the two true utilities ($\boldsymbol{u}(\boldsymbol{x}_{\boldsymbol{i}})$ and $\boldsymbol{u}(\boldsymbol{x}_{\boldsymbol{j}})$). Because of the assumption of constant noise, the probability of choosing $\boldsymbol{x}_{\boldsymbol{i}}$ over $\boldsymbol{x}_{\boldsymbol{j}}$ would be directly proportional to the distance between the true utility of two options. In accordance with McFadden’s formulation (McFadden 1974; 2005; Stott 2006), we assumed that the distribution of error differences ($\varepsilon_{j} - \varepsilon_{i}$) took a logistic form:

$P(x_{i}) = \frac{1}{(1+e^{-\Delta utility})}$ Eq. 6

and then used the inverse of this logistic distribution’s CDF to estimate the difference in utilities ($\Delta utility$) between the hypothetical 0.03 ml reward gaps (Fig. 3c) - essentially the slope of the utility function at every midpoint. The cumulative sum of these slopes provided an estimate of the utility at each midpoint.

### Estimating utility functions from risky and riskless choices in a common metric

Because the utilities measured from aggregate behavior did not account for probability weighting on choices (i.e. they were EUT utilities rather than PT values), parametric utility functions were re-estimated from individual choices using a discrete choice model that could account for the effects of both, separately. As in most discrete choice models (and in line with the aggregate RUM metric), a logit function (softmax) was used to represent noise in the decision-making process. The probability of the monkey making either a left or right choice was therefore given by:

$P_{chooseLeft} =\frac{1}{\left( 1+e^{-\lambda\left( V_{Left}-V_{Right}- \theta\right)} \right)}$ Eq. 7

where the probability of choosing the left option is a function of the difference in value between the left and right options, the noise parameter, $\lambda$, and the side bias parameter $\theta$. The value of each option (V_Left_, V_Right_) took on the functional form prescribed by PT in its cumulative form (Tversky & Kahneman 1992):

$V\left( m_{1}{,m}_{2},p_{2} \right)= u\left( m_{2} \right)*w\left( p_{2} \right)+u\left( m_{1} \right)*(1-w\left( p_{2} \right))$ Eq. 8

where *m_1_* and *m_2_* were the low and high outcome magnitudes respectively, while *p_2_* was the probability of obtaining the high outcome; the probability weighting function (*w(p)*) corresponded to a power function:

$w\left( p \right) =p^{\rho}$ Eq. 9

For risky choices, a 1-parameter power function captured the weighting of probabilities (Eq. 9). A $\rho$ < 1 implied an overweighting of the probability of receiving the highest reward whilst $\rho$ > 1 implied underweighting. The utility of the option’s outcome (*u(m)*) was the CDF of a two-sided power distribution (Kotz and Dorp 2010):

$u(m) = \left\{ \begin{matrix} \kappa\left( \frac{m}{\kappa} \right)^{1/\alpha} & for 0\leq m\leq\kappa\\ 1- (1-\kappa)\left( \frac{1-m}{1-\kappa} \right)^{1/\alpha} & for \kappa<m\leq1 \end{matrix} \right.$ Eq. 10

In the probability weighting function, the ρ-parameter implied either an overweighing (ρ < 1) or underweighting (ρ > 1) of an outcome’s probability. The utility measure was a function of two parameters: the α parameter represented the non-linearity of the utility function: s-shaped (α < 1), inverse-s-shaped (α > 1) or linear (α = 1); the $\kappa$parameter represented the inflection point where the curvature of the utility function would invert. Each outcome magnitude (*m*) was normalized onto a 0-1 scale, so that κ was bounded by the range of outcome magnitudes experienced by the monkeys (values from 0 to 1, corresponding to 0 ml and 0.5 ml respectively).

In our discrete choice model, utility functions took the form of the cumulative distribution function of a two-sided power distribution (Eq. 10; Kotz & Dorp 2010), a 2-parameter function that could easily account for complex risk-attitudes (Kontek and Lewandowski 2018): if $\alpha$ < 1, the utility function would be convex and predict risk-seeking choices up to the inflection at parameter $\kappa$ (predicting risk-averse choices thereafter); if instead $\alpha$ > 1, the utility function would be concave and predict risk-averse behavior up to the inflection at $\kappa$ (predicting risk-seeking behavior afterwards). Each of these parameters was fit to single-choice data by maximizing the sum of log-likelihoods defined on the model as:

$LL(\theta| y) =\sum_{i = 1}^{n} y_{i}*\log\left( P_{Choose Left} \right)+\sum_{i = 1}^{n} y_{i}^{'}*\log\left( 1- P_{Choose Left} \right)$ Eq. 11

For each individual choice trial (*i*), *y* and *y’* indicated a left or right choice respectively (1 if yes, 0 if no), n was the total number of trials for the session, and $P_{Choose Left}$ was the output of the earlier logistic function (Eq. 7). This discrete choice analysis was restricted to choice sequences previously deemed appropriate for the aggregate preference estimations described in earlier sections.
